# Supplementary material for: Umbilical cord separation time, predictors and healing complications in newborns with dry care
Source: PLoS One. 2020 Jan 10;15(1):e0227209. doi: 10.1371/journal.pone.0227209 (PMC6953818; doi:10.1371/journal.pone.0227209)
Supplement: Attachment — Submitted filename: Response to Reviewers.docx [file pone.0227209.s002.docx]

**Response to Reviewers:**

Dear Anna Palatnik, Academic Editor of PLOS ONE and anonymous reviewers:

After a careful review of our proposed article, based on the suggestions made by the reviewers, we sent it for further evaluation. In the new manuscript, we have highlighted in yellow color the modifications made to the original text.

We would like to express our sincere thanks to the reviewers for their great work; their annotations have allowed us to improve the manuscript.

Here is how, we have responded to the reviewers' suggestions in the new version of our proposed article. We hope that the work done will achieve the final approval of the Editorial Team of Plos One. If not, all authors are at your disposal to resolve any issues or proceed with further revisions as necessary.

Kind regards,

The authors.

**Journal Requirements:**

- A revision of the style of the manuscript has been carried out according to the requirements of Plos One.
- Submitted the minimum set of anonymous data needed to replicate study findings as a supporting information file.
- Included subtitles for the Supporting Information files at the end of the manuscript, and updated citations in the text to match accordingly.

**Reviewers' comments:**

- **Reviewer #1:**
  - Comment 1: “The authors chose to study this issue prospectively and compare their rates of infection and other complications. This topic has been extensively investigated before by other groups but this group effectively describe their concerns about parental refusal to believe these recommendations. Studying this issue in their region could help the doctors in that region families to follow the stated guidelines”.
    - Response 1:

The study population is reluctant to change, most of the research available in the scientific literature is from other regions, and this study will help us provide evidence-based care and improve understanding of the mistrust of families in our study area.

- Comment 2: “The authors represent the time of separation is different ways in the abstract vs manuscript which was a bit confusing (I would recommend using days rather than seconds)”.
  - Response 2:

Umbilical cord separation time changed in the manuscript and in the abstract using days, to make it understandable.

- **Reviewer #2:**
  - Comment 1: “The World Health Organization is written as both the World Health Organization and the World Health Organisation”.
    - Response 1:

Corrected grammatical errors; the World Health Organization name changed and others minor grammatical errors.

- Comment 2: “some of the phrasing is a little awkward, especially when
  using passive voice”.
  - Response 2:

Modified part of the phrasing from passive voice to simple past in the manuscript:

- Line 72: Omphalitis is an infection of the umbilical cord stump.
- Line 83-85: Estimated that the incidence of omphalitis in newborns in developed countries is around 0.7% and rises to 6–8% in developing countries.
- Line 159-162: The different actions carried out in the dry umbilical cord care: how often the umbilical cord gets wet, pH of soap used, type of bath for neonates, wearing a garment that tightens the newborn's body, placing the newborn's diaper under the umbilical cord, and covering the umbilical cord with clean gauze.
- Line 169: In table 1, we can see the independent secondary variables of the studied population.
- Line 225-226: Jaén Research Ethics Committee (26 November 2015) approved the study.
- Line 261: In table 3, we can see the correlation between umbilical cord care and separation time.
- Line 407-408: other authors obtained similar results [30].
- Line 417-418: Literature supports the umbilical cord separation time predictors reported by our research.
- Comment 3: “In particular, omphalitis can have considerable morbidity and mortality, a risk that increases if the umbilical cord remains attached and theoretically is then reduced if the umbilical cord detaches more quickly. This is seen in line 66 and 67. Consider including the reference for this statement that connects the time of umbilical cord attachment and increased risk of omphalitis, especially if there is any statistical information where a particular length of time increases the risk of omphalitis by a known amount. This would help further support the overall clinical importance of this research because even though the time of cord separation was statistically different in some cases this might not result in a clinically significant length of time to the neonate”.
  - Response 3:

We have added a reference to lines 66-68. A possible consequence of bacterial colonisation is cord stump infection, a factor that can greatly increase morbidity and mortality. The risk of infection increases until the stump detaches [4].

- Comment 4: *“*For example, though there is a clear statistical difference of about 2 days between the groups of neonates whose umbilical cords became wet 0-4 times vs. >5 times, does 2 days make a clinical significance to these children? However, if the aim was merely to show that the clean and dry method is both safe and financially sound then the current information provided would be sufficient*”*.
  - Response 4:

We provide this information to know the factors involved in the procedure of the fall of the umbilical cord. There are no statistical data in the scientific literature to support our results.

- Comment 5: “Please discuss the training or role of the researcher who is taking the measurements for the umbilical cord and providing a clinical assessment/
  possible diagnosis of omphalitis. Are they a physician? Nurse?”
  - Response 5:

We specify in several parts of the article who does the clinical assessment (line 150,156,178).

- Comment 6: “In line 151, please provide the definition that the paper uses for late separation of the umbilical cord”.
  - Response 6:

In line 151 (actually 153-156) we add a bibliographic reference that provides the definition for late umbilical cord separation.

- Comment 7: “On page 8, within the socio-demographic and clinical newborn variables, please list gestational age as a length of weeks and days. For example, 40 weeks and 0 days instead of 280 days”.
  - Response 7:

On page 8 we change the duration of gestational age to express it in weeks and days.

- Comment 8: “State clearly that the umbilical cord separation time of interest in your research is the time when the umbilical cord naturally separates from the infant. This will clear up any questions that the reader might have when reading the title or abstract and wondering if the authors meant the time of clamping the cord from the mother at birth (delayed cord clamping) or something even more extreme such as the lotus birth where the placenta is never clamped and remains attached to the infant for several days”.
  - Response 8:

In Line 148, 149 we add the definition of umbilical cord separation time: Umbilical cord separation time. Is time when the umbilical cord naturally separates from the infant.
